# Supplementary figures and images for: Comprehensive multi-omics analysis reveals the prognostic and immune regulatory characteristics of the PTPN family in osteosarcoma
Source: PLoS One. 2025 Jun 26;20(6):e0326872. doi: 10.1371/journal.pone.0326872 (PMC12200852; doi:10.1371/journal.pone.0326872)

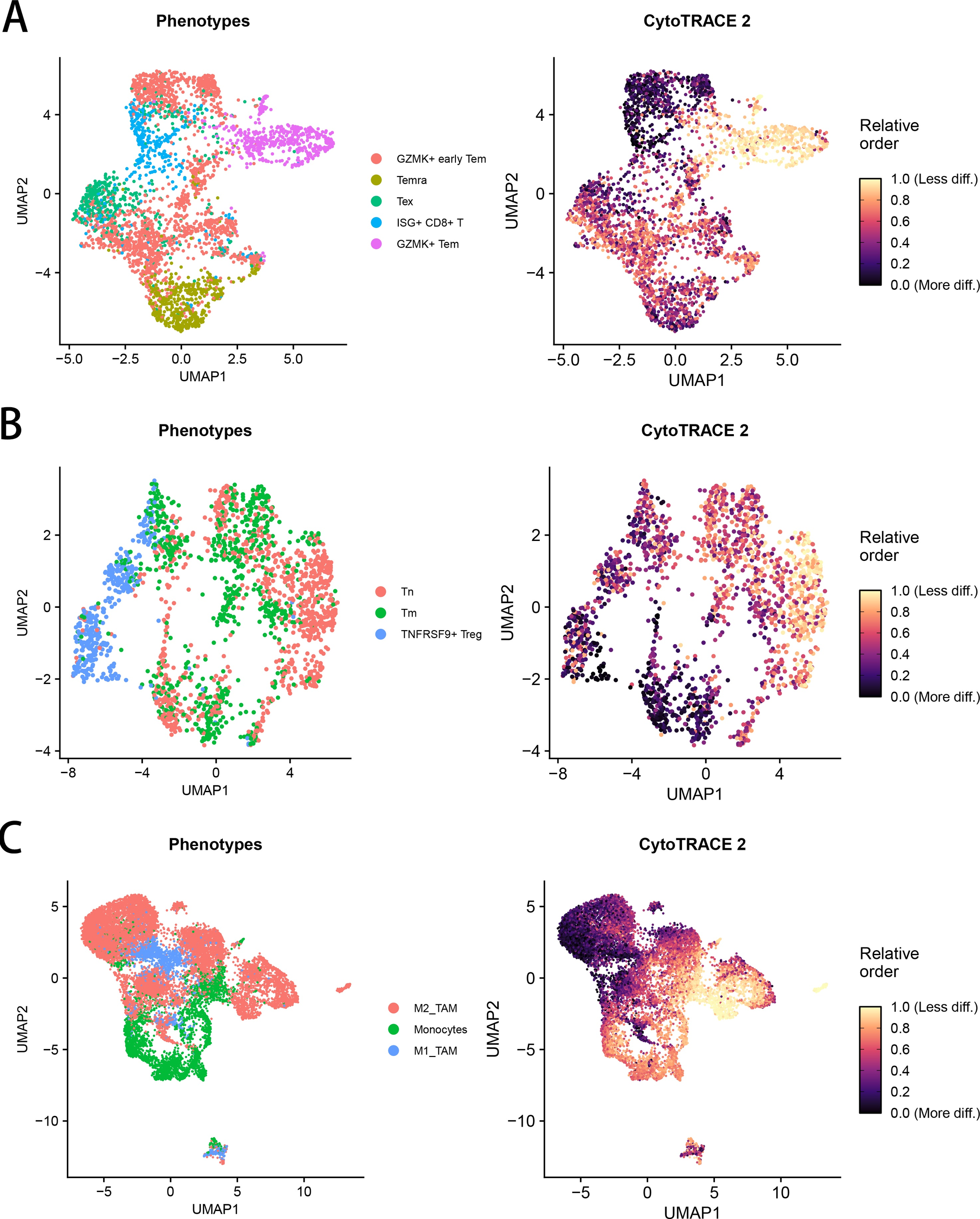

Supplement: S1 Fig — (A) UMAP of mononuclear macrophages and T cell phenotypes (GZMK+ early Tem, Temra, Tex, ISG + CD8 + T, GZMK+ Tem) with CytoTRACE values indicating differentiation states. (B) UMAP of CD4 + T cells, showing naive (Tn), memory (Tm), and TNFRSF9 + Treg populations, along with corresponding CytoTRACE differentiation levels. (C) UMAP of mononuclear phagocytes (M2-TAM, monocytes, M1-TAM) with CytoTRACE values illustrating their differentiation states. (TIF) [file pone.0326872.s001.tif]

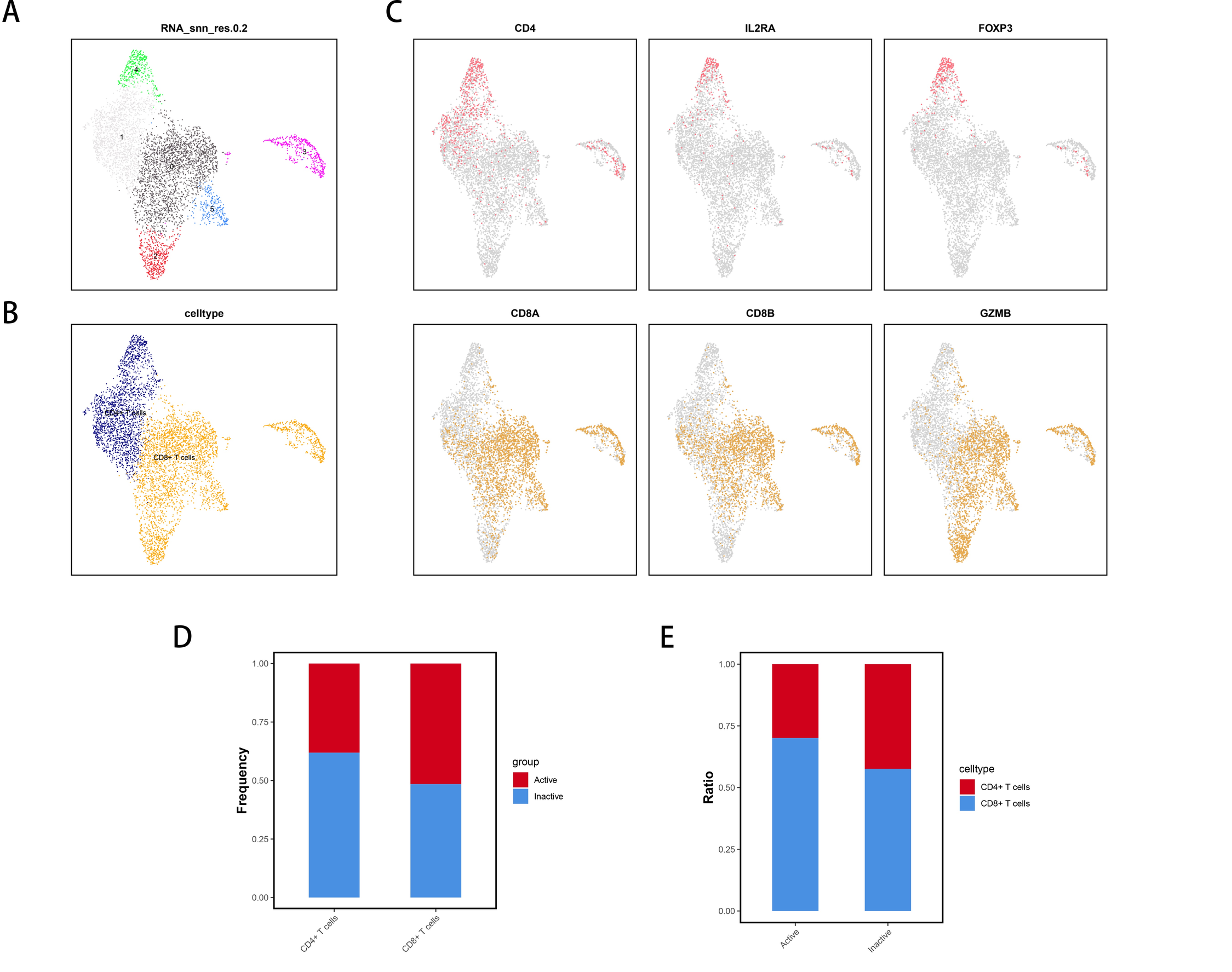

Supplement: S2 Fig — (A) UMAP visualization showing clustering of CD4+ and CD8 + T cells based on RNA expression profiles, with distinct color coding for different cell populations. (B) Cell type annotation indicating CD4 + T cells (in yellow) and CD8 + T cells (in blue), highlighting their distribution within the UMAP. (C) Expression patterns of key markers (CD4, IL2RA, FOXP3, CD8A, GZMB) across the cell populations, with color intensity reflecting marker expression levels. (D) Bar graph illustrating the frequency of Active (red) and Inactive (blue) states for CD4+ and CD8 + T cells. (E) Bar graph showing the ratio of Active and Inactive states for CD4+ and CD8 + T cells, indicating overall proportions between the two cell types. (TIF) [file pone.0326872.s002.tif]

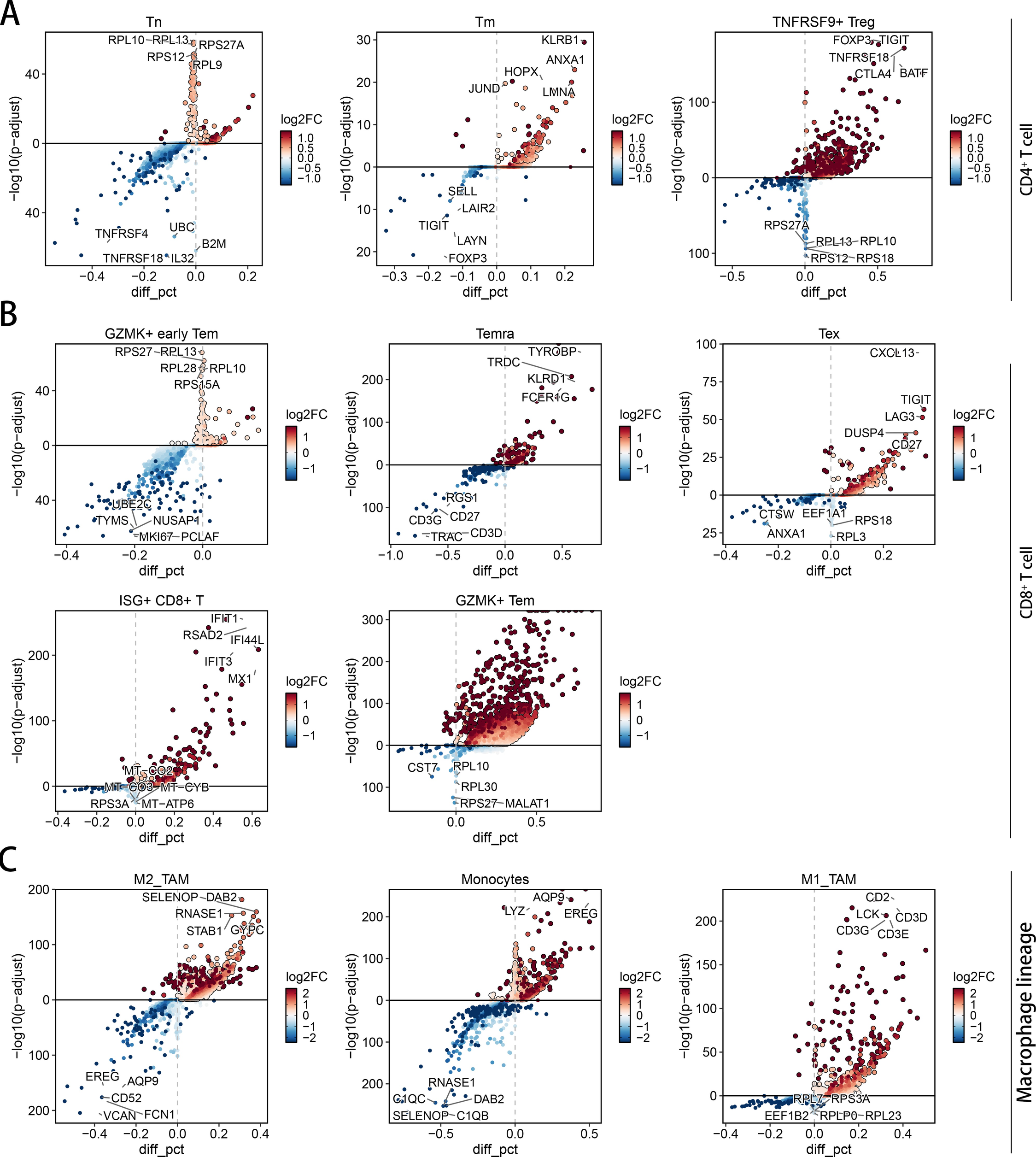

Supplement: S3 Fig — (A) Volcano plots showing differentially expressed genes for CD4 + T cell subsets, including naive (Tn), memory (Tm), and TNFRSF9 + Treg cells, with key genes highlighted. (B) Volcano plots for CD8 + T cell populations, showcasing GZMK+ early Tem, Temra, Tex, ISG + CD8 + , and GZMK+ Tem subsets, emphasizing significant gene expression changes. (C) Volcano plots representing mononuclear macrophage populations, including M2_TAM, monocytes, and M1_TAM, identifying key genes that define their transcriptional profiles. (TIF) [file pone.0326872.s003.tif]

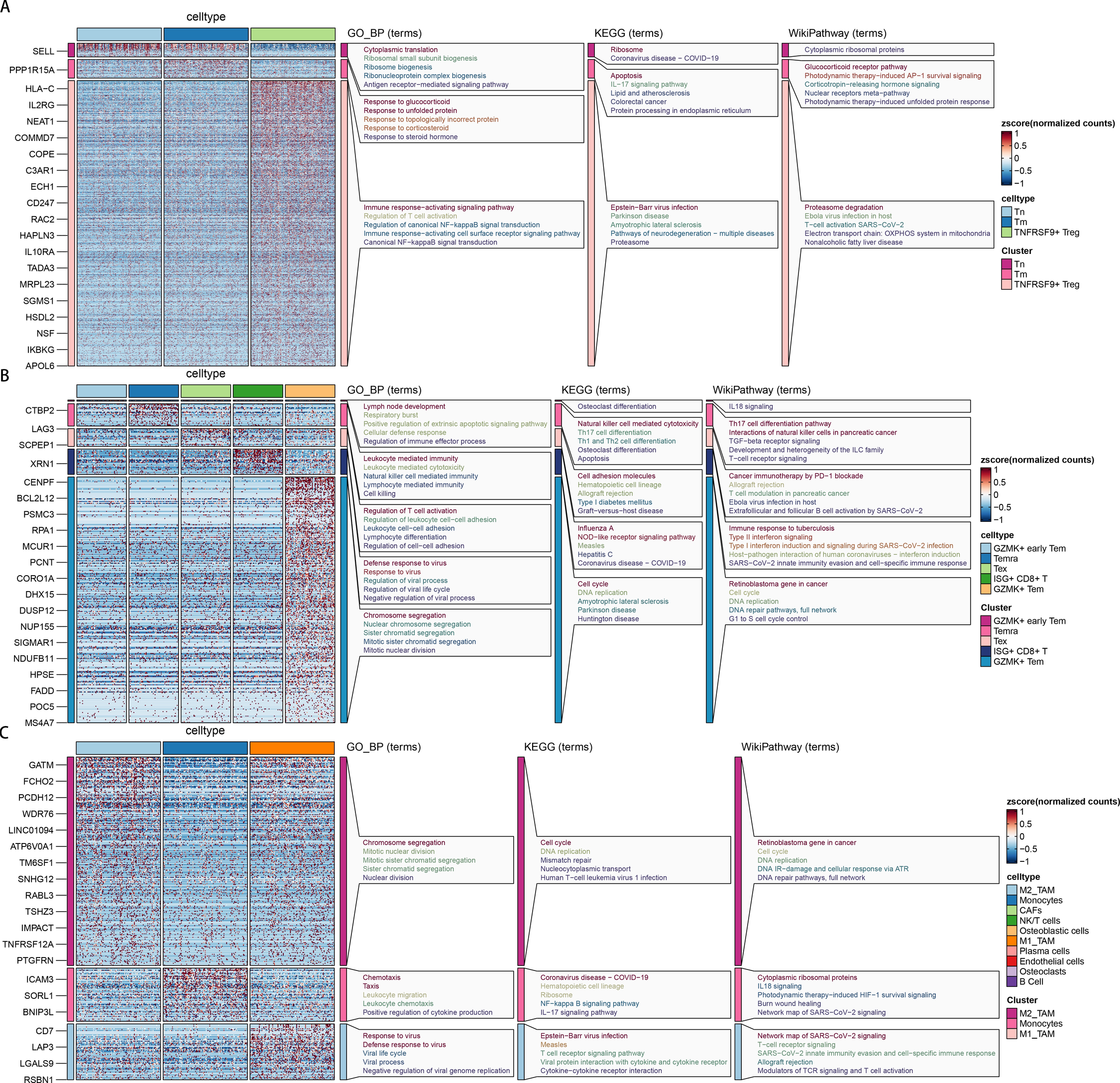

Supplement: S4 Fig — (A) Heatmap and enrichment analysis of gene expression for mononuclear macrophages, highlighting key biological processes, pathways, and enriched terms associated with their activity. (B) Heatmap and enrichment analysis for CD4+ T cell subsets, including naive (Tn), memory (Tm), and TNFRSF9+ Treg cells, showcasing significant gene expression and enriched pathways relevant to their functions. (C) Heatmap and enrichment analysis of CD8+ T cell populations, featuring GZMK+ early Tem, Temra, Tex, ISG+ CD8 + , and GZMK+ Tem subsets, illustrating differences in gene expression and pathway enrichment critical for their immune roles. (TIF) [file pone.0326872.s004.tif]

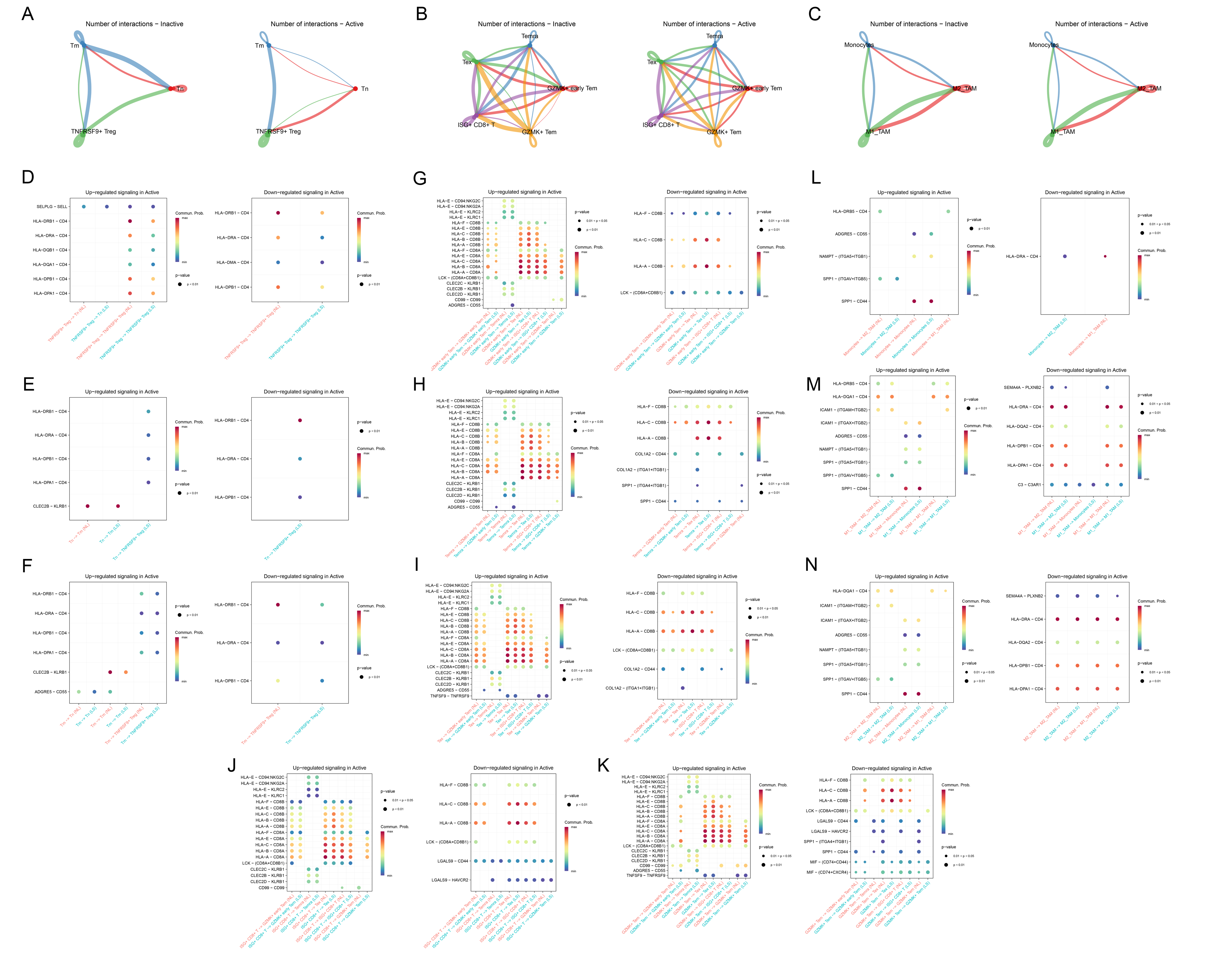

Supplement: S5 Fig — (A-C) the interactions among different cell types under these two conditions. (D-F) Scatter plots showing the upregulated and downregulated ligands and receptors for naive T cells, memory T cells, and regulatory T cells. Additionally, panels (G-K) Scatter plots that depict the upregulated and downregulated ligands and receptors for GZMK+ early memory T cells, TEMRA, GZMK+ memory T cells, exhausted T cells, and ISG+ CD8+ T cells. (L-N) The upregulated and downregulated ligands and receptors for monocytes, M1 tumor-associated macrophages (TAMs), and M2 TAMs. (TIF) [file pone.0326872.s005.tif]

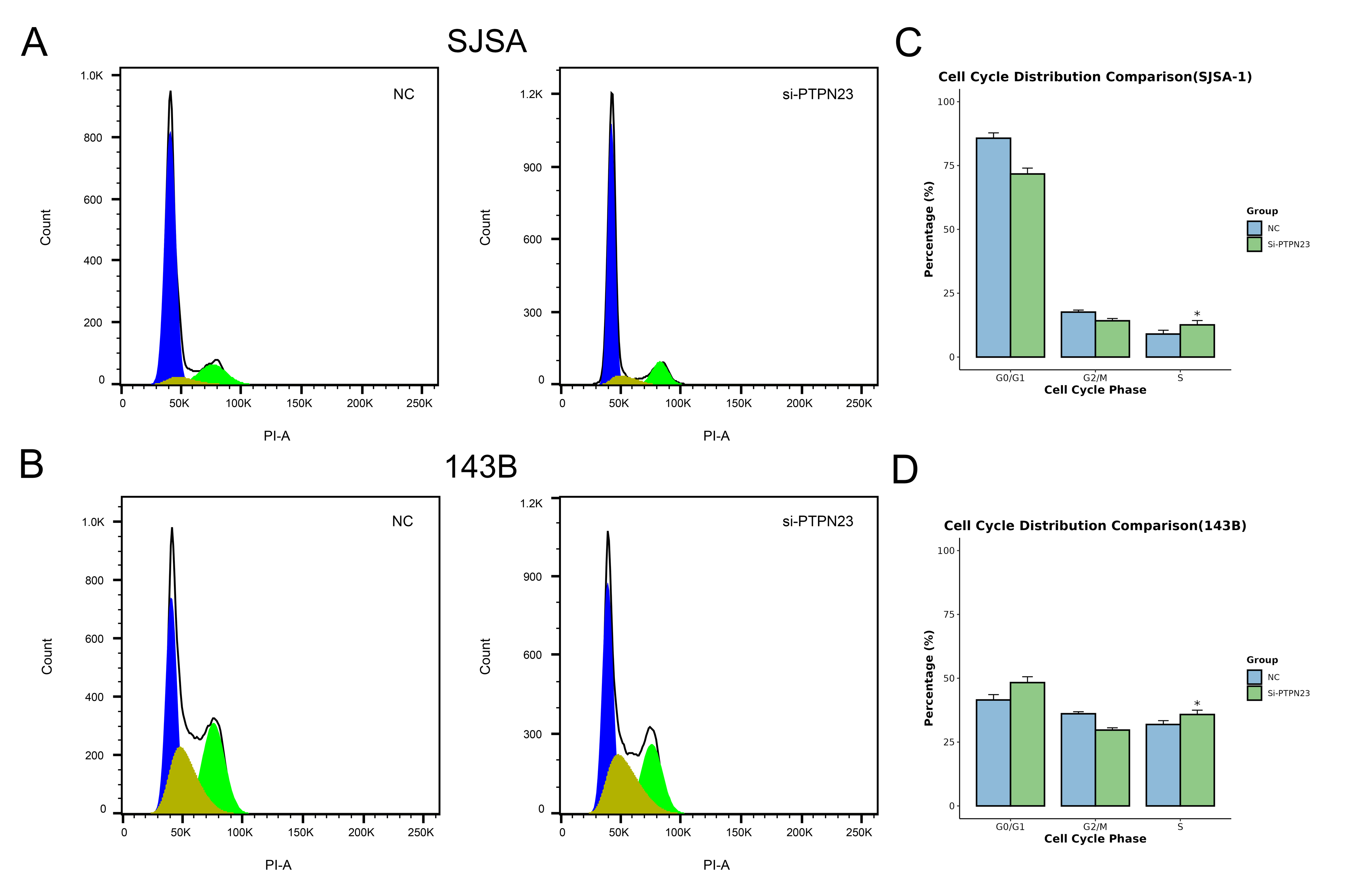

Supplement: S6 Fig — (A-B) Flow cytometric analysis of the cell cycle distribution in SJSA-1 and 143B cells. The left panel represents the negative control (NC), while the right panel shows the results for si-PTPN23 treatment. The different phases of the cell cycle are indicated by the various colors: G0/G1 (blue), S (green), and G2/M (orange). (C-D) Bar graph comparing the cell cycle phase distribution in SJSA-1 and 143B cells between the NC and si-PTPN23 groups. The percentage of cells in G0/G1, S, and G2/M phases is shown, with asterisks indicating statistical significance (*p < 0.05). (TIF) [file pone.0326872.s006.tif]

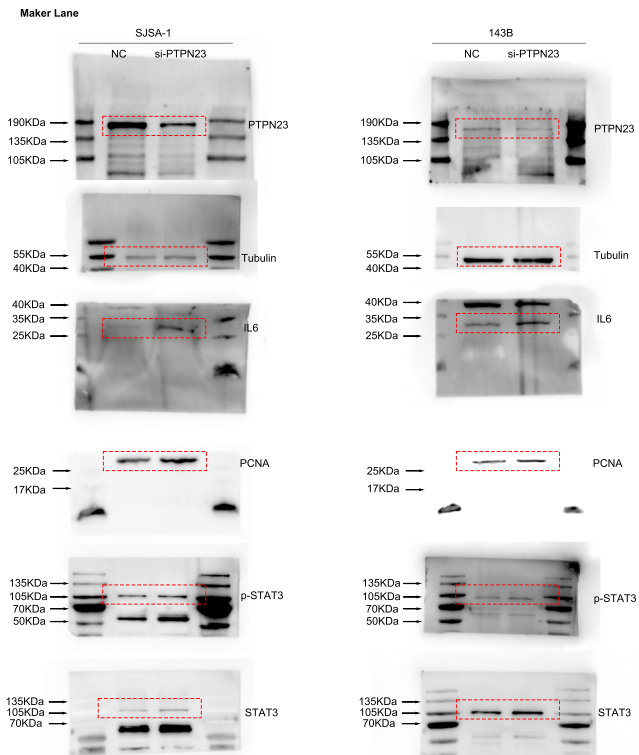

Detection:ECL

Supplement: S8 Raw images — (PDF) [file pone.0326872.s008.pdf]
