# Supplementary material for: Comprehensive multi-omics analysis reveals the prognostic and immune regulatory characteristics of the PTPN family in osteosarcoma
Source: PLoS One. 2025 Jun 26;20(6):e0326872. doi: 10.1371/journal.pone.0326872 (PMC12200852; doi:10.1371/journal.pone.0326872)
Supplement: Attachment — Submitted filename: Response to Reviewers 1.docx [file pone.0326872.s010.docx]

Response to Reviewers 1

Reviewer #1: The authors have carried out a series of studies on osteosarcoma using multi-omics analysis, which is quite interesting. There is few mistake in the manuscript and the experimental results show good and new results. So I recommend to you that this manuscript should receive revision. The following are the questions and some mistakes in this manuscript:
1.It is noted that your manuscript needs careful editing by someone with expertise in technical English editing paying particular attention to English grammar, spelling, and sentence structure so that the goals and results of the study are clear to the reader.

Answer：Thank you for your feedback. I have carefully revised the manuscript to address issues related to grammar and sentence structure throughout the text, with specific changes highlighted in red.
2.All the blots/gels must have the molecular weight/bp indicated.

Answer：We have added the molecular weight/bp information in Fig 8D.
3.Include scale bars to all microscopy images.

Answer：I have modified Fig 8C to include images captured at a scale of 200 µm.
4.Small interfering RNA (siRNA) sequences need to be clearly listed.

Answer：Thank you for your valuable feedback. I have clearly listed the small interfering RNA (siRNA) sequences in the Methods section, specifically on lines 145-148.
Although the author conducted experiments for verification, I think the simple in vitro cell experiment is still not convincing enough, and I suggest adding in vivo experiments to further verify the relevant conclusions.

Answer：Thank you for your insightful comment. I appreciate your suggestion regarding the inclusion of in vivo experiments to strengthen our findings. While we believe that the in vitro results provide important preliminary data, we acknowledge the value of in vivo validation. We will consider this recommendation for future work to enhance the robustness of our conclusions.
