# Supplementary material for: Comprehensive multi-omics analysis reveals the prognostic and immune regulatory characteristics of the PTPN family in osteosarcoma
Source: PLoS One. 2025 Jun 26;20(6):e0326872. doi: 10.1371/journal.pone.0326872 (PMC12200852; doi:10.1371/journal.pone.0326872)
Supplement: Attachment — Submitted filename: Response to Reviewers 2.docx [file pone.0326872.s011.docx]

Reviewer #2: We thank the authors for their submission, and recognize the hard work they put in preparing this manuscript for submission. It is a thorough study and seeks to leverage a large amount of data in order to identify possible therapeutic targets in osteosarcoma. There are, however, areas which need to be addressed to help the authors obtain their own goal within the current scope of this project to impact their field as they seek to do:

1. While attention has been paid to grammar and editing the English used in this manuscript, it would be worth having a native English speaker take a pass at ending some of the phrases used. Lines such as “The KEGG analysis” on line 196 and use of “based on your findings” on line 200 stand out as easily addressable grammar issues.
Answer：Thank you for reviewing our manuscript and for your valuable feedback. We appreciate your attention to the language and grammar aspects of our work. In response to your comments, we have thoroughly revised the entire manuscript for grammar corrections. We have highlighted all changes in red for your convenience. Additionally, we sought input from a native English speaker to ensure the clarity and fluency of our expressions.

2. We appreciate the authors dual focus on T cells and macrophages and the role PTPNs may play in controlling osteosarcoma outcomes in their patient population. To that end, the data focuses on the differentiation from naïve T cells present in the samples into memory and regulatory T cells. However, the authors only use single biomarkers to identify these cell types and do not compare the subgroups across memory and regulatory T cells to see if there is an impact of the tumor microenvironment on their development. A fuller perspective on which T cell populations are affected by the tumor and its impact on cellular PTPN levels would be far informative for the reader. Similar questions may be asked of the monocyte-macrophage aspect of the study, as well.
Answer：Thank you for your valuable feedback and for highlighting the importance of our focus on T cells and macrophages in relation to PTPNs and osteosarcoma outcomes.

In response to your concerns regarding the identification of T cell populations and their differentiation, we have conducted further analyses using cell communication methodologies (Cellchat). This approach allowed us to examine the signaling interactions between CD4+ T cells, CD8+ T cells, and monocyte-macrophages, specifically analyzing the ligands and receptors involved in these interactions. Additionally, detailed information on CD4+ T cells (lines 339-349), CD8+ T cells (lines 377-385), and monocyte-macrophages (lines 413-425) is included in the manuscript. The corresponding visualizations can be found in S5 Fig. We believe that these enhancements provide a fuller perspective on how the tumor microenvironment influences the development of these T cell populations and their associated cellular PTPN levels. We appreciate your suggestions and believe that these revisions will make the manuscript more informative and comprehensive for the readers.

3. The authors describe the use of gene enrichment to highlight PTPN family gene significance in active versus inactive gene signatures in Figure 3. However, what is lacking is a clear description of the active versus inactive signatures are, how they were validated in this disease type, and whether the genes identified in this study align with previous reports.

Answer：Thank you for your constructive feedback regarding our analysis of PTPN family gene significance in active versus inactive gene signatures.

To differentiate between active and inactive signatures, we performed AUCell analysis on the genes PTPN1 to PTPN23, as illustrated in Fig. 3B. This methodology is detailed in lines 102-103 of the manuscript. Additionally, we conducted pseudobulk analysis along with differential expression and functional enrichment analyses, with results presented in Fig. 3G-H. A thorough description of these analyses and their validation within the context of osteosarcoma is provided in lines 291-295.

Moreover, we discussed the relevance of PTPN23 in relation to other cancers in lines 565-578 of the manuscript. Our findings indicate that PTPN23 has been identified as a tumor suppressor gene in various cancer types, aligning with observations in the existing literature.

4. There is a distinct lack of control in the proliferation studies performed in this study, making it hard to derive validity and impact of the si-PTPN23 transfection into both osteosarcoma cell lines. Is the lack of proliferation due to cell death or simply paused by inhibition of PTPN23? The authors should consider using a non-dye based assay or measure cell viability at these time points to strength the data set.
Answer：Thank you for your thoughtful comments regarding our proliferation studies involving si-PTPN23 transfection in osteosarcoma cell lines. In our experiments, we conducted small interfering RNA (siRNA) transfection targeting PTPN23 in osteosarcoma cell lines. To ensure proper controls, we used a transfection reagent for both the treatment group (si-PTPN23) and the control group (si-NC), as clearly indicated in Fig. 8. Additionally, to improve clarity regarding our grouping conditions, we provided detailed descriptions in lines 462-465 of the manuscript.

To further investigate the specific effect of si-PTPN23 on cell proliferation, we performed cell cycle analysis using flow cytometry. Our findings indicated an increase in the S phase after si-PTPN23 treatment, suggesting enhanced cellular proliferation. This is detailed in Supplementary Figure S6, with further discussion provided in lines 466-471 of the manuscript. Regarding your concerns about the perceived lack of cell proliferation, we observed in our cell cycle analysis that the proportion of cells in the S phase increased following si-PTPN23 treatment, indicating that cell proliferation is indeed promoted under conditions of PTPN23 suppression.

5. Echoing the previous manuscript review, the authors have a good opportunity to validate these findings with in vivo experiments. It would be beneficial to see them propose a key and informative next step which they are pursuing along this route of investigation to link with future work.

Answer：Thank you for your constructive feedback. I would like to emphasize that we recognize the need for further experiments to deepen our understanding of these findings. We have outlined our plans for additional studies in lines 589-596 of the manuscript.

6. In addition to discussing future experiments, the authors have the opportunity to propose a PTPN centric mechanism that is driving the disease course and what inhibition of PTPN23 could mean for downstream cell signaling and immune effector responses based off the data presented. It would strengthen the conclusions of this study and

Answer：Thank you for your insightful feedback and suggestions. In response to your comments regarding the proposal of a PTPN-centric mechanism driving disease progression and the potential therapeutic strategies targeting PTPN23, we have addressed these points in lines 596-604 of the manuscript.
